# Automatic classification of focal liver lesions based on MRI and risk factors

**DOI:** 10.1371/journal.pone.0217053

**Published:** 2019-05-16

**Authors:** Mariëlle J. A. Jansen, Hugo J. Kuijf, Wouter B. Veldhuis, Frank J. Wessels, Max A. Viergever, Josien P. W. Pluim

**Affiliations:** 1 Image Sciences Institute, University Medical Center Utrecht & Utrecht University, Utrecht, the Netherlands; 2 Department of Radiology, University Medical Center Utrecht, Utrecht, the Netherlands; University of Braunschweig - Institute of Technology, GERMANY

## Abstract

**Objectives:**

Accurate classification of focal liver lesions is an important part of liver disease diagnostics. In clinical practice, the lesion type is often determined from the abdominal MR examination, which includes T2-weighted and dynamic contrast enhanced (DCE) MR images. To date, only T2-weighted images are exploited for automatic classification of focal liver lesions. In this study additional MR sequences and risk factors are used for automatic classification to improve the results and to make a step forward to a clinically useful aid for radiologists.

**Materials and methods:**

Clinical MRI data sets of 95 patients with in total 125 benign lesions (40 adenomas, 29 cysts and 56 hemangiomas) and 88 malignant lesions (30 hepatocellular carcinomas (HCC) and 58 metastases) were included in this study. Contrast curve, gray level histogram, and gray level co-occurrence matrix texture features were extracted from the DCE-MR and T2-weighted images. In addition, risk factors including the presence of steatosis, cirrhosis, and a known primary tumor were used as features. Fifty features with the highest ANOVA F-score were selected and fed to an extremely randomized trees classifier. The classifier evaluation was performed using the leave-one-out principle and receiver operating characteristic (ROC) curve analysis.

**Results:**

The overall accuracy for the classification of the five major focal liver lesion types is 0.77. The sensitivity/specificity is 0.80/0.78, 0.93/0.93, 0.84/0.82, 0.73/0.56, and 0.62/0.77 for adenoma, cyst, hemangioma, HCC, and metastasis, respectively.

**Conclusion:**

The proposed classification system using features derived from clinical DCE-MR and T2-weighted images, with additional risk factors is able to differentiate five common types of lesions and is a step forward to a clinically useful aid for focal liver lesion diagnosis.

## Introduction

Proper differentiation between benign and malignant liver lesions is relevant to avoid unnecessary biopsies. Additionally, characterization and classification of focal liver lesions is of great importance for adequate treatment. An MRI examination of the liver, including a dynamic contrast enhanced (DCE) series, is an effective tool for detection and characterization of liver lesions by radiologists, thanks to the good soft tissue contrast. For example, hyper intensity on T2-weighted MR images, enhancement patterns on DCE-MR images and the presence of washout of contrast agent are features used in clinical practice to differentiate focal liver lesions[1, 2]. Furthermore, clinical meta information and risk factors, such as symptoms, age, sex, and a known primary tumor, help in decision making[3, 4]. Nonetheless, it can still be difficult and time consuming to distinguish lesion types, because of the broad range of lesion appearances on MRI.[5] An automatic classification system could aid radiologists in this task.

Some efforts have been made to automatically classify focal liver lesions using T2-weighted MR images to aid radiologists. Mayerhoefer et al. (2010)[6] applied texture-based classification on T2-weighted and T1-weighted MR images, to distinguish cysts and hemangiomas. This classification system reached accuracy rates of 0.77–0.84 for T1-weighted images and 0.75–0.88 for T2-weighted images, for differentiating two benign lesion classes. Another classification system based on features derived from T2-weighted MR images, developed by Gatos et al. (2017)[7], differentiates between benign lesions, hepatocellular carcinoma (HCC) within a cirrhotic liver, and metastases within a noncirrhotic liver. This system reached an overall accuracy of 0.90. However, it was not designed to distinguish different types of benign lesions. Both methods applied features derived from T2-weighted images, but did not include risk factors or features derived from DCE-MR images, while these features may supply additional information for a classification system to differentiate more lesion types[4, 8].

This paper proposes a classification method that aims to differentiate between five lesion classes: adenomas, cysts, hemangiomas, HCCs and metastases by exploiting features derived from DCE-MR images with an extracellular contrast agent, as well as features from T2-weighted images. Risk factors for adenoma, HCC and metastasis were also taken into account as features.

## Materials and methods

### Data

The study comprises MRI data of patients with suspicion of liver lesions from the University Medical Center Utrecht, The Netherlands, acquired between February 2015 and February 2017. The UMCU Medical Ethical Committee has reviewed this study and informed consent was waived due to its retrospective nature. Patients without lesions, with lesions other than the common adenomas, cysts, hemangiomas, HCCs or metastases, or with atypical lesions were excluded. Focal nodular hyperplasias (FNHs) were not included, because there was an insufficient number of FNHs for training a classifier. Also, liver lesions with a diameter of less than 5 mm were excluded from this study. Up to four lesions per patient were included. In order to balance the classes, the data sets from patients with liver metastases acquired between February 2015 and February 2016, were excluded. In total, 95 patient data sets with 125 benign lesions (40 adenomas, 29 cysts and 56 hemangiomas) and 88 malignant lesions (30 HCCs and 58 metastases) were included in this study. The origin of the primary tumor of the metastases was widespread, including: 8 breast carcinomas, 47 gastrointestinal carcinomas (23 colorectal carcinomas, 18 neuroendocrine carcinomas, 4 esophagus carcinomas, 1 HCC metastasis, and 1 adenocarcinoma), and 3 have another primary tumor origin. The metastases were therefore of both hypervascular (25) and hypovascular (32) type[1].

### MR imaging

**Fig 1:**
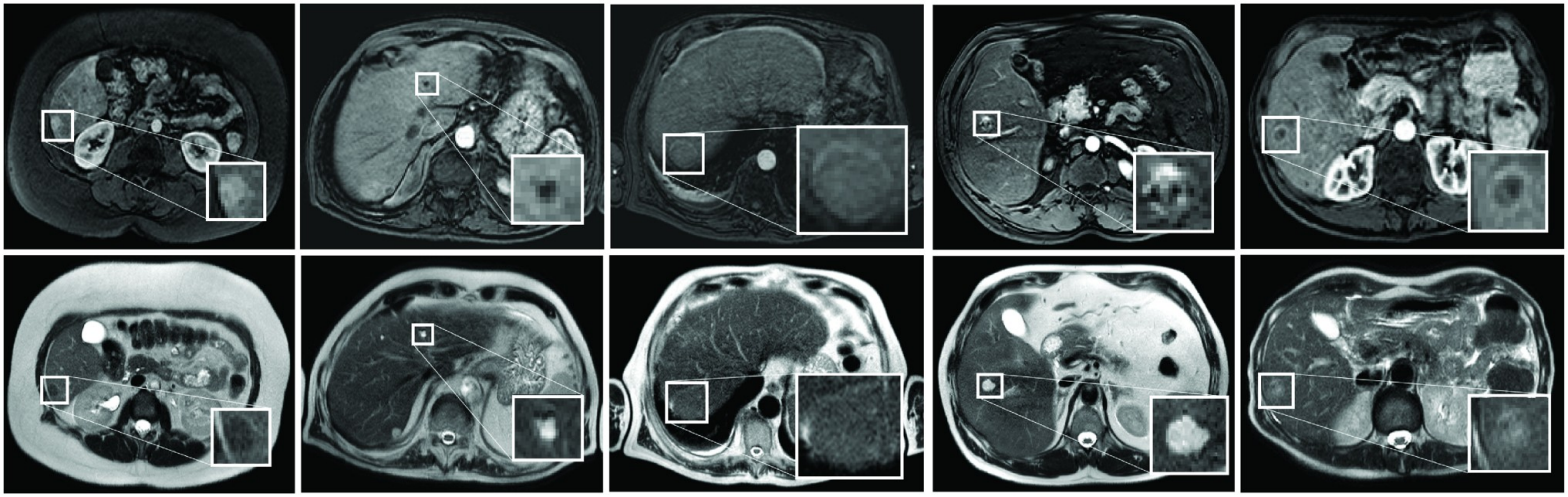
Examples of focal liver lesions. From left to right livers with an adenoma, cyst, HCC, hemangioma, and a metastasis from colorectal carcinoma origin are shown. The top row shows the arterial phases of the DCE-MRI and the bottom row the T2-weighted images. A zoom-in of the lesions is inserted.

All 95 patients had an MRI examination on a 1.5 T scanner (Philips) with the clinical focus on the liver, including a DCE-MRI and a T2-weighted scan. Examples of lesions in DCE-MRI and T2-weighted images are shown in Fig 1. The DCE-MR images were acquired in six breath holds with one to five 3D images per breath hold. The DCE-MRI series was acquired using a clinical protocol with the following parameters: TE: 2.143 ms; TR: 4.524 ms; flip angle: 10 degrees. After acquiring the first image, gadobutrol (0.1 ml/kg Gadovist of 1.0 mmol/ml at 1 ml/s) was administered at once, followed by 25 ml saline solution at 1 ml/s. In total, 16 3D images per patient were acquired with 100–119 slices and matrix sizes ranging from 256 × 256 to 288 × 288. Voxel size was 1.543 mm × 1.543 mm × 2 mm. The T2-weighted images were acquired during free breathing using a TSE protocol with the following parameters: TE: 80 ms; TR: 756 ms; flip angle: 90 degrees. Matrix sizes ranged from 448 × 448 to 512 × 512, with 25–30 slices. Voxel size was 0.938 mm × 0.938 mm × 8 mm. Eight patients underwent a slightly modified T2-weighted acquisition due to a change in the clinical scanning protocol, with: TE: 102 ms; TR: 2945 ms. The slice thickness became 5 mm, resulting in 41 slices for the same FOV. Two other patients were scanned with a fat-suppressed T2-weighted SPAIR protocol, in which the voxel size was 0.893 mm × 0.893 mm × 5 mm and the matrix size was 448 × 448 with 46 slices. All scans were acquired in axial direction.

The DCE-MR series were corrected for motion using a PCA-based groupwise registration algorithm[9] as described in Jansen et al. (2017)[10]. The intensities in the DCE-MR series were linearly remapped between 0 (background) and 1 (contrast agent peak in the aorta) for standardization. For the T2-weighted images the original intensities were kept, because the highest intensity is dependent on the type of lesion, i.e. cysts can have a higher intensity than the gallbladder or the spinal fluid.

All lesions were manually segmented on a single slice in both the motion-corrected DCE-MR series and the T2-weighted images by a researcher and were verified by an independent expert. Malignant lesions were additionally proven by pathology or follow-up. However, for lesions classified by an expert radiologist as benign, a follow-up was not available. The area of the lesion segmentations ranged from 0.2 to 53.0 cm^2^ in the 2D slice, with a median of 1.8 cm^2^. Furthermore, a small part, on average 3.5 cm^2^, of healthy parenchyma was manually segmented in the DCE-MR series for feature calculation.

### Feature extraction

In total, 164 features were extracted from the DCE-MR and T2-weighted MR images within the lesion, including contrast curve features, gray level histogram features, and GLCM texture features. Part of the features were chosen based on characteristics used by radiologists during visual rating. The other part of the feature categories were already proven to be of value in the classification of liver lesions [6, 7] or breast lesions with DCE-MR imaging [11, 12]. Risk factors, as the presence of steatosis, cirrhosis, and the presence or absence of a known primary tumor somewhere in the body, were also used as features. An overview of all features is given in Table 1. Below we will explain how the contrast curves, images, and histograms are defined, on which the features are calculated.

**Table 1:** Features derived from DCE-MR and T2-weighted images and risk factors.

Categories	Features	Calculated on:
Contrast curve features	Maximum enhancement, time to peak (TTP), uptake rate, washout rate [11], area under the curve (AUC), average plateau, early-to-late signal enhancement ratio (SER) and time of arrival[12]	TIC, CEC, CE lesion-parenchyma-ratio, TIC lesion-parenchyma ratio
Gray level histogram features	Mean, standard deviation, skewness, kurtosis and the 10^th^ and 90^th^ percentile of the intensities	Pre-contrast image, TTP image, and late enhancement phase of the DCE-MR series, T2-weighted image, TTP feature map, radial gradient histogram of late arterial enhancement phase[13], ring enhancement histogram of portal-venous phase.
Texture features (gray level co-occurrence matrix (GLCM) features)	Angular second moment, contrast, correlation, sum of squares variance, homogeneity, sum average, sum variance, entropy, sum entropy, difference variance, difference entropy, IMC1 and IMC2; calculated from the summed gray level co-occurrence matrix (GLCM) in 4 directions, 0°, 45°, 90° and 135°, with an offset of 1 pixel	Pre-contrast image, TTP image, and late enhancement phase of the of DCE-MR series, variance in all DCE-MR images of the series, T2-weighted image, TTP feature map
Risk factors and other	Presence of steatosis, cirrhosis, and primary tumor in the body, area of lesion	

TIC = time intensity curve, CEC = contrast enhanced curve, CE = contrast enhanced.

#### Contrast curves

The contrast curve features were obtained from the lesion time intensity curve (TIC) and three curves derived from it. To smooth the TIC, the motion-corrected DCE-MR series were first filtered with the TIPS bilateral filter[14]. The TIC was obtained for each pixel in the lesion mask and in the healthy parenchyma mask. The three other contrast curves are the contrast enhancement curve (CEC), the contrast enhancement (CE) lesion-parenchyma ratio, and the TIC lesion-parenchyma ratio. The CEC was calculated by dividing the lesion TIC by the intensity of the pixel in the pre-contrast image. The CE lesion-parenchyma ratio was obtained by dividing the average CEC of the lesion ROI time point-wise by the average CEC of the parenchyma ROI, which was calculated in a similar manner as the CEC of the lesion. The TIC lesion-parenchyma ratio was obtained by dividing the average TIC of the lesion ROI time point-wise by the average TIC of the parenchyma ROI. The average curves were obtained by taking the average for each time point within the lesion and were used to calculate the features, as given in Table 1. For the CEC and the CE lesion-parenchyma ratio curves, the standard deviation of the feature values within the lesion was included as feature.

#### Images and histograms

**Fig 2:**
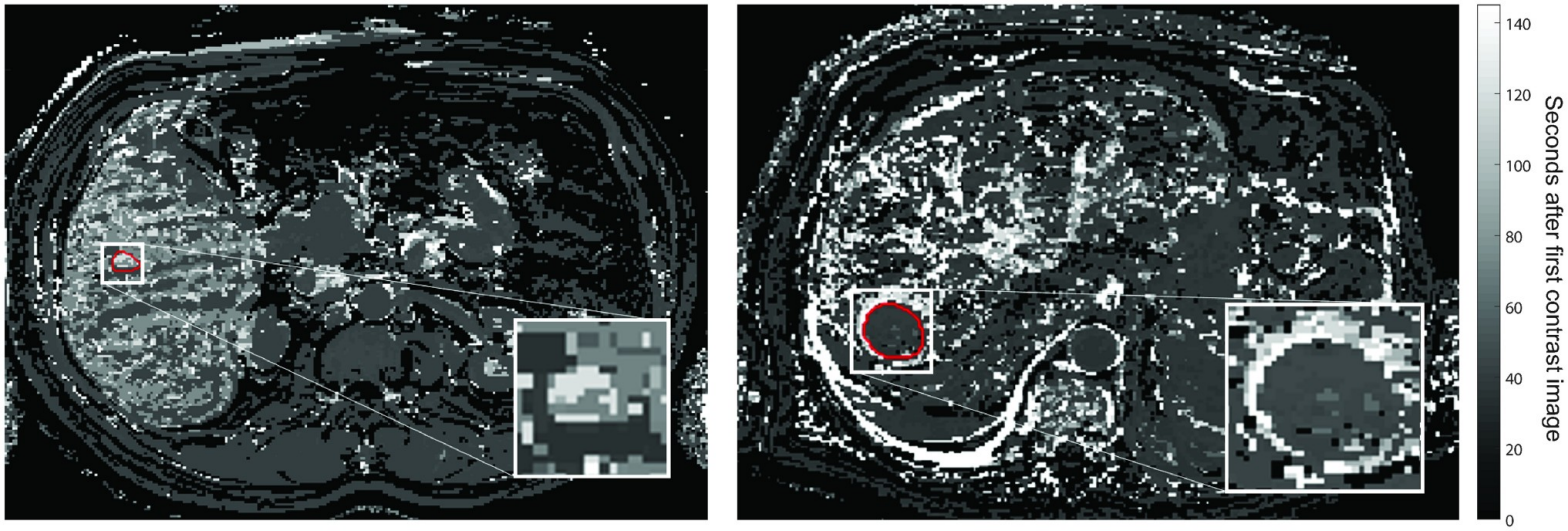
Time-to-peak feature maps of a liver with a hemangioma (left) and a HCC (right). The lesions correspond with the lesions in Fig 1. The red contours show the lesion segmentations.

Gray level histogram features and GLCM texture features were derived from the pre-contrast image, the time-to-peak (TTP) image and late arterial enhancement phase image of the DCE-MR series, as well as from the T2-weighted image and the TTP feature map. The variance of the GLCM texture features throughout the DCE-MR series was also included. The TTP image was defined as the image of the DCE-MR series in which the maximum enhancement of the lesion in the average CE curve was reached. The TTP feature map was defined as the TTP value in seconds obtained per voxel. Two examples of TTP feature maps are shown in Fig 2.

Additionally, histogram features were calculated from the radial gradient histogram and the ring enhancement histogram. The radial gradient histogram was obtained from the late arterial enhancement phase, multiplying the difference in intensity between each voxel value and the lesion’s central voxel value with the distance between this voxel and the central voxel. The values are then normalized between 0 and 1. The features derived from this histogram provide information about the circularity of the enhancement within the lesion. Mean values around 1 indicate a homogenous spherical pattern in the lesion[13]. The ring enhancement histogram was obtained by calculating the normalized distances between the center of the lesion and the pixel with the maximum first derivative of the portal-venous phase intensities, in all radial directions. The first derivatives were calculated between the central voxel and the edges of the lesion segmentation mask plus two extra voxels. The portal-venous phase would show the ring enhancement if present. A mean ring enhancement of 1 indicates ring enhancement and a high SD indicates heterogeneous enhancement.

#### Risk factor and other features

If the presence of steatosis was stated in the clinical report, the corresponding feature was assigned the value one, in absence the value zero. The same was done for the presence of cirrhosis and for the presence of a primary tumor. The area of the lesion was equal to the number of pixels within the lesion mask times the pixel area.

### Classification

An extremely randomized trees classifier[15] was chosen for classification, as it is a non-parametric, robust classifier that assembles decision trees using all available training samples at a low computational cost. At each node of a tree, a random set of thresholds is applied to a random subset of the features. The feature with the best threshold is selected for that node. In this way the most informative features are mostly used in the nodes of the trees. The usage of the extremely randomized tree classifier makes the need for the optimal feature selection less strict. To avoid overfitting a minimum of five samples per leaf was set, feature selection was performed, and the number of features offered to each node in the classifier was equal to the square root of the total number of features.

The classification system was implemented using scikit-learn[16]. A built-in function for feature selection applying the ANOVA F-score is used to exclude the features without any information regarding the classification of the liver lesions.

## Experiments

The experiments are split into two parts. First we will demonstrate the impact of adding features from DCE-MR images and risk factors to the T2-weighted MR images features by training the extremely randomized trees classifier on different feature sets, in a similar way as was reported by Jansen et al. (2018)[17]. Secondly, we focus on the optimization and classification of the lesion using all features.

In this part of the study, feature set A) contained only the features from T2-weighted MR images, feature set B) contained only the features from T2-weighted MR images and the risk factor features, feature set C) contained the features from T2-weighted MR images and DCE-MR images, and feature set D) contained all the available features. For a fair comparison, only the 19 features with the highest ANOVA F-score were selected, because the smallest feature set (A) only comprises 19 features. The number of features offered to each node in the classifier was equal to four, the square root of the total number of features; in total 700 trees were built. Sensitivity, specificity, and accuracy are calculated based on a leave-one-patient-out cross-validation. The leave-one-patient-out cross-validation was selected to be able to have enough training samples for generalization of the large variations in the data. The results are compared for the classification of the five lesion classes.

For the second part of this study, the optimal number of features and trees for the classifier were determined during an optimization process using cross-validation with the number features ranging from 10 to 164, in steps of 10, and the number of trees ranging from 100 to 800, in steps of 50. In this way, features that do not hold any information for the classification of these liver lesions are excluded. For this classification problem 50 features and 700 trees were optimal and used for the next experiments. The number of trees had only a small impact on the classification results, while the number of features should be chosen more carefully. Too many features could lead to a large amount of non-informative trees and overfitting.[18]

Feature selection was performed on all features to remove redundant features and speed up the classification. The 50 features with the highest ANOVA F-score were selected for classification. The number of features offered to each node in the classifier was equal to seven, the square root of the total number of features; in total 700 trees were built. The classes were balanced per sample.

The classification system was evaluated by calculating the sensitivity, specificity and the overall accuracy based on a leave-one-patient-out principle. Feature selection was repeated during the leave-one-patient-out evaluation to avoid a positive bias. Additionally, for each of the five classes, a one-versus-others accuracy was calculated by splitting the confusion matrix in one lesion class versus the other lesion classes. The receiver operating characteristic (ROC) curve is also obtained for each lesion class, showing the strength of the classifier for individual lesion classes. The ROC curve for a particular lesion class was calculated by adding the probabilities of the other four lesion classes as other-class. The optimal cut-off values and the corresponding true positive rate (TPR), false positive rate (FPR) and false negative rate (FNR) were obtained for each lesion type using these ROC curves.

The overall accuracy, sensitivity and specificity for differentiating benign and malignant lesions was calculated by separating benign and malignant lesions in the confusion matrix. Furthermore, the ROC curve of benign-versus-malignant lesions was obtained by plotting the added probabilities of the benign lesions versus those of the malignant lesions. Using this ROC curve the optimal cut-off value and the corresponding TPR, FPR and FNR were obtained.

## Results

The results of the first part of the experiments are presented in Table 2, showing the classification results for the different feature sets. The results show that in general the sensitivity, specificity and overall accuracy increase, when DCE-MR image features, risk factor features, or both are added to the T2-weighted MR image features. All feature sets B, C, and D improve significantly over feature set A, using a Chi-Square McNemar test. Therefore, all the features were used to train an extremely randomized trees classifier with an optimal number of features.

**Table 2:** Classification results for the four different feature sets (sensitivity/specificity), with 19 features selected.

	A	B	C	D
Adenoma	0.43 / 0.50	0.68 / 0.75	0.65 / 0.65	0.50/ 0.64
Cyst	0.76 / 0.79	0.72 / 0.75	0.93 / 0.90	1.00 / 0.91
Hemangioma	0.77 / 0.75	0.73 / 0.73	0.79 / 0.81	0.75 / 0.82
HCC	0.57 / 0.36	0.73 / 0.58	0.63 / 0.56	0.77 / 0.55
Metastasis	0.41 / 0.51	0.60 / 0.64	0.69 / 0.73	0.66 / 0.67
**Overall accuracy**	**0.58**	**0.69**	**0.73**	**0.71**

A) T2-weighted MR features, B) T2-weighted MR features and risk factor features, C) T2-weighted MR and DCE-MR features, D) all features.

The remainder of this section shows the results of the second part of the experiments, based on all features (feature set D). Table 3 shows the 42 features that are selected in every leave-one-patient-out repetition. Four features that are selected in >90% of the leave-one-patient-out repetitions are also included in Table 3 and indicated with an asterisk. In addition, three features are selected in >75% of the leave-one-patient-out repetitions: the sum of squares variance of the TTP image, and the standard deviations of the gray level histogram of the TTP feature map and the radial gradient histogram. The less frequently selected features (<50%) are: the presence of cirrhosis, the presence of a primary tumor in the body, the IMC2 of the pre-contrast image, and standard deviation of the gray level histogram of the late enhancement image. Additionally, from the TTP image the sum average, sum variance, and IMC1 were selected less frequently.

**Table 3:** Selected features with the highest ANOVA F-scores.

Contrast curve features	Gray level histogram features	Texture features	Risk factors and other
TTP *CEC lesion-parenchyma ratio*	Mean, 10^th^ perc., 90^th^ perc. *Pre-contrast image*	SSVar, SumVar*, IMC1 *Pre-contrast image*	Presence of steatosis
Max. enhancement, SER, AUC *TIC*	Mean, SD*, 10^th^ perc., 90^th^ perc. *TTP image*	SSVar, sum average, SumVar *Late enhancement image*	
Max. enhancement, TTP, uptake*, average plateau, AUC *TIC lesion-parenchyma ratio*	Mean, 10^th^ perc., 90^th^ perc. *Late enhancement image*	Correlation, SSVar, sum average, SumVar, sum entropy, DiffVar, IMC 1 and 2 *T2*-*weighted image*	
	Mean, SD, skewness, 10^th^ perc., 90^th^ perc. *T2*-*weighted image*	Contrast*, SSVar, SumVar, DiffVar *TTP feature map*	
	Mean, 90^th^ perc. *TTP feature map*		

TTP = time to peak; SER = early-to-late enhancement ratio; AUC = area under the curve; SSVar = sum of squares variance; SumVar = sum variance; DiffVar = difference variance. The input image/histogram of the features is listed in italic print. The asterisks indicate a feature selected in >90% of the leave-one-patient-out repetitions.

The confusion matrix with the sensitivity and specificity per class, together with the overall accuracy is presented in Table 4. The one-versus-others accuracy is given per lesion class. The overall accuracy is 0.77.

**Table 4:** Confusion matrix of the five class problem, including the sensitivity, specificity and one-versus-other accuracy per lesion class.

	Adenoma	Cyst	Hemangioma	HCC	Metastasis	Sens	Spec	One-vs-other accuracy
Adenoma	32	0	2	4	2	**0.80**	**0.78**	**0.92**
Cyst	0	27	2	0	0	**0.93**	**0.93**	**0.99**
Hemangioma	3	2	47	0	4	**0.84**	**0.82**	**0.91**
HCC	3	0	0	22	5	**0.73**	**0.56**	**0.88**
Metastasis	3	0	6	13	36	**0.62**	**0.77**	**0.85**
**Overall accuracy: 0.77**								

The rows represent the true class and the columns represent the predicted class.

**Fig 3:**
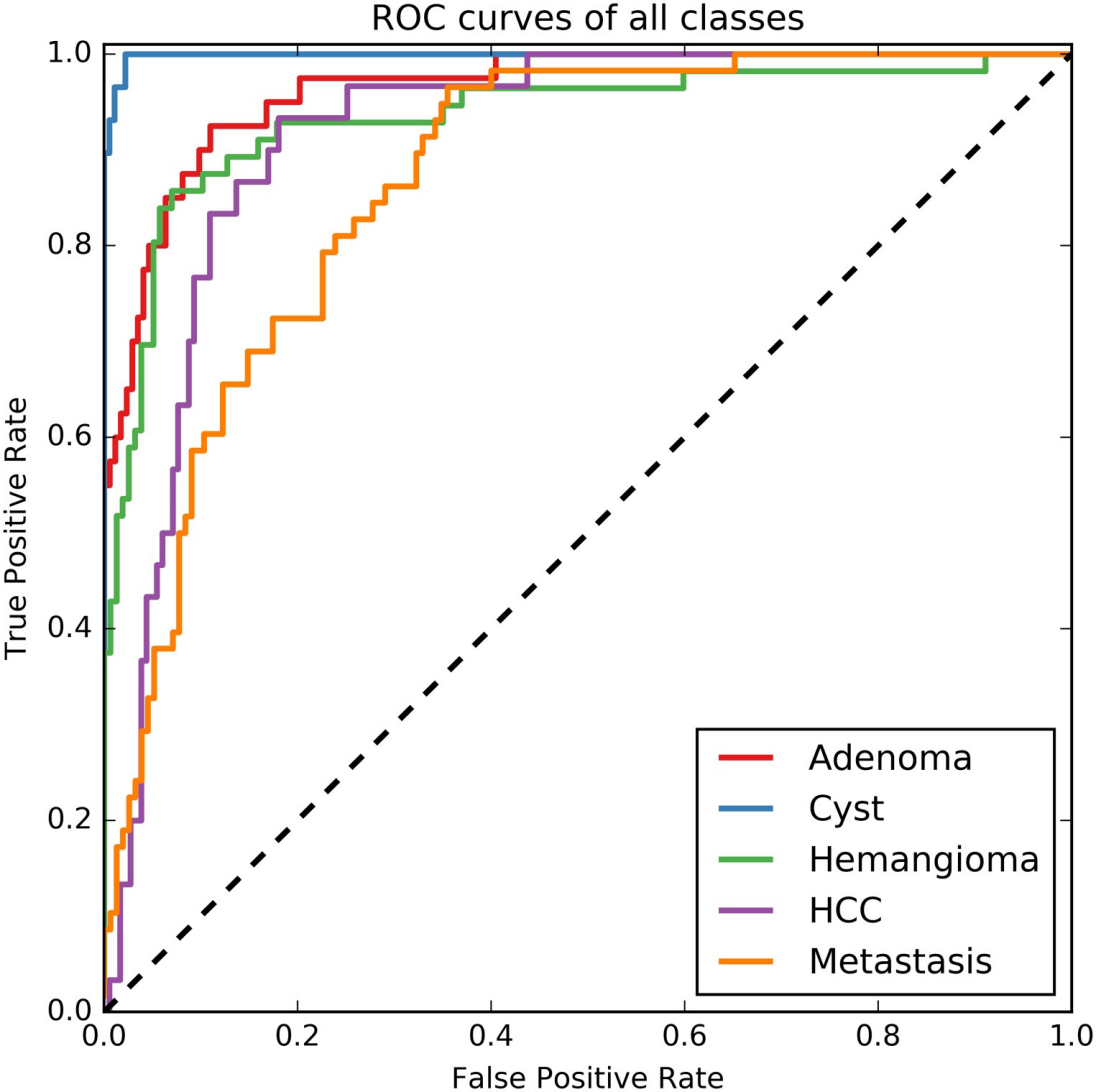
ROC curves of all lesion classes in a one-versus-other approach. The rest class is calculated as the outcome probabilities of the other four lesions.

The ROC curves for each of the five lesion classes are shown in Fig 3. In Table 5 the area under the ROC curve (AUC) is given, together with the optimal cut-off values for each individual lesion class and the corresponding TPR, FPR and FNR.

**Table 5:** Areas under the ROC curve (AUC) for each class including the optimal cut-off value and the corresponding true positive rate (TPR), false positive rate (FPR) and false negative rate (FNR).

	AUC	Optimal cut-off value	TPR	FPR	FNR
Adenoma	0.96	0.28	0.88	0.08	0.12
Cyst	1.00	0.43	0.97	0.01	0.03
Hemangioma	0.93	0.26	0.89	0.13	0.11
HCC	0.91	0.33	0.83	0.11	0.17
Metastasis	0.88	0.26	0.81	0.24	0.19

### Benign versus malignant lesions

**Fig 4:**
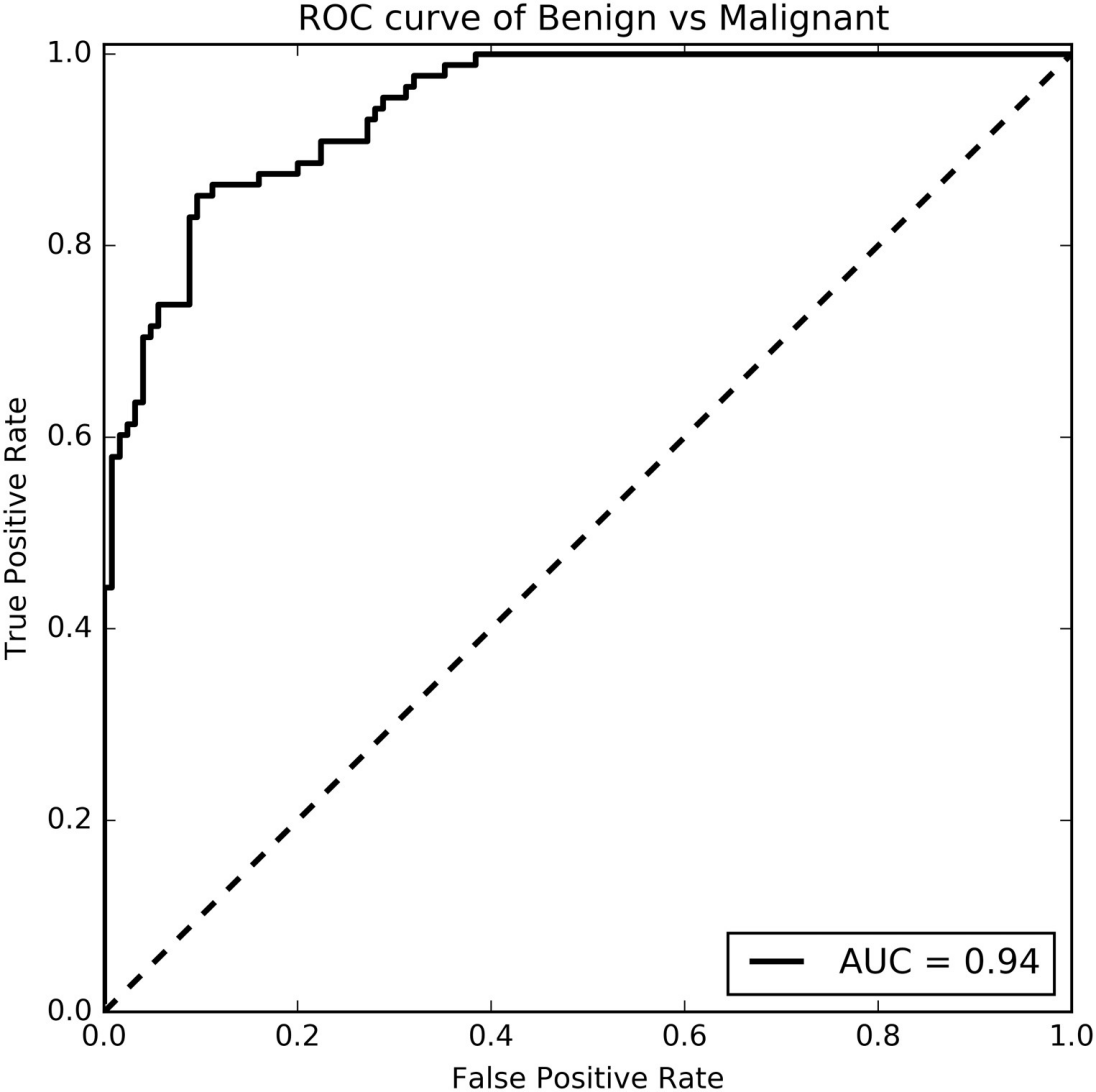
ROC curve of benign-versus-malignant classification problem. The area under the ROC curve (AUC) is 0.94.

Splitting the lesions in the confusion matrix in benign lesions (adenoma, cyst and hemangioma) and malignant lesions (HCC and metastasis), gives a sensitivity of 0.92 for benign lesions and 0.86 for malignant lesions. The specificity is 0.91 and 0.88 for benign and malignant lesions respectively and the overall accuracy is 0.90. The ROC curve for the benign-vs-malignant lesions is shown in Fig 4. The classifier gives a TPR of 0.89, a FPR of 0.20 and a FNR of 0.11 for the malignant lesions at the optimal cut-off value of 0.42. The area under the ROC curve is 0.94 for the benign-versus-malignant classification problem.

## Discussion

In this study, a classification system was proposed based on features derived from T2-weighted and DCE-MR images, and from risk factors. The classification system was able to differentiate five lesion types: adenoma, cyst, hemangioma, HCC, and metastasis, with an overall accuracy of 0.77. The average sensitivity [min-max] is 0.79 [0.62–0.93] and the average specificity [min-max] is 0.77 [0.56–0.93].

The classification system was challenged by the fact that the lesion types had a wide range of appearances and thus a large variance in feature values. For example, smaller HCCs and hemangiomas have a different appearance than larger ones on both DCE-MR and T2-weighted MR images[5, 19]. Furthermore, there was a large variety in the origin of the primary tumor of the metastases, but all these metastases were treated as one lesion type, leading to some variety in lesion appearance. The large variance in appearances leads to overlapping feature values between lesion types. Nonetheless, the classifier was able to correctly determine the lesion type in the vast majority of cases. The unequal distribution of the origin of the primary tumor did not seem to have an effect on the classification results. When we separately analyze the three major origin types (colorectal carcinomas, neuro-endocrine tumors, and breast carcinomas), they have similar sensitivities. Only cysts have very typical features in both T2-weighted MR and DCE-MR images, and were therefore the only lesions with a small variance in feature values, resulting in the highest sensitivity and specificity.

The area under the ROC curve for each of the individual lesion types is high, ranging from 0.86 to 1.00, implying that the classifier is highly capable of distinguishing one lesion type from the others. The optimal cut-off values in these ROC curves are low in comparison to what one might expect in a two-class problem, but in this study the probabilities of the other four lesions are added instead of running the classifier again on the 2-class problem. The TPR and the FPR result from selecting the lesion type for which the probability of that lesion exceeds the optimal cut-off value as given in Table 5, instead of selecting the lesion type with the highest probability, as was done for Table 4. The assignment of a lesion type based on the optimal cut-off value results in the possibility that one lesion could have been assigned two labels. For this reason the FPR and TPR results of Table 5 do not match the sensitivity and specificity results from the confusion matrix in Table 4.

The overall accuracy for benign and malignant lesions, when splitting the confusion matrix of Table 4, is 0.90. This is higher than the five lesion types’ classification, largely due to the mix up of metastases and HCC, both malignant lesions. The classifier is thus not only able to differentiate five types of lesions, but is also able to differentiate benign from malignant lesions with an even higher accuracy, even when it is not trained for this classification problem.

Training the classifier on four different feature sets shows that both risk factor features and DCE-MR image features greatly improved the classification results, despite the fact that the number of features is suboptimal for feature set C and D. Only for hemangiomas and cysts did the addition of risk factor features not improve the classification results for the suboptimal feature set.

Unfortunately, no public data set for this application is available, but we compare the proposed method with a recent study by Gatos et al. (2017)[7] by performing the evaluation in a similar way. The study by Gatos et al. (2017) reported an overall accuracy of 0.90 for the classification of benign, HCC and metastatic focal liver lesions using only T2-weighted MR images. Our classifier was not able to obtain high accuracies with only T2-weighted MRI features, possibly because our data set included different types of lesions and has more classes than the study by Gatos et al. (2017). Nonetheless, similar one-vs-other accuracies per class were reported for benign, HCC, and metastasis classes, when exploiting features from T2-weighted MR images, DCE-MR images and risk factors.

Even though not all T2-weighted MR images in this study were acquired using the same protocol, the classifier was able to learn to correctly identify the lesions independent of small changes in the T2-weighted protocol used. This shows that the classifier is robust to slight changes in certain features.

The feature selection procedure was performed separately for every leave-one-patient-out repetition, to avoid any positive bias. Out of all repetitions, 42 features were selected in every repetition and four in >90% of the repetitions. This might indicate that the core set of 42 features are important for the classification task.

The selected features originate from all four feature categories and from DCE-MR and T2-weighted images, which underpins the importance of a wide selection of features. The optimization process for the number of features and trees in the extra trees classifier showed that the number of features had a larger impact than the number of trees. Yet, the number of trees should be sufficient for each feature to be selected in the nodes of the trees at least a few times. Having too many (redundant) features, i.e. more than 100 features, as input for the classifier will lead to overfitting and decreases the accuracy with a few percentages.

### Limitations

Five common lesion types were included in this study, which does not cover the full range. Lesion types with very low occurrence in the dataset could not be included in the classification. A larger data set with a vast amount of samples per lesion type would be needed to train a classification system that is able to differentiate more lesion types with a better accuracy.

Feature calculations were done in 2D, because this reflected how the manual annotations were made and provided to the classifier. If the final method were to be applied in a clinical workflow, being able to perform classification based on a 2D lesion annotation is beneficial over requiring 3D lesion annotations. The latter are more time-consuming to create. Automatic 3D lesion segmentation, if available, would enable 3D feature calculations, which potentially further improves the performance of our proposed method.

Moreover, lesions smaller than 5 mm in diameter were excluded because texture features could not be reliably computed on lesions smaller than 3×3 pixels. Doing the calculations in 3D might overcome part of this problem.

### Future work

The features in this study are based on lesion characteristics used by radiologists in clinical practice. Although the features are carefully designed, they might not be the best representation of the focal liver lesions. Learning features from the data instead of using hand-crafted features might improve the classification results. Unsupervised feature learning algorithms in combination with a supervised classifier have been used for classification of lesions in other applications.[20] For example, restricted Boltzmann machines and convolutional sparse auto-encoders have successfully been applied as feature extractors and combined with machine learning classifiers[21, 22]. In future work, the possibilities of such representation learning algorithms to automatically generate a feature set with a better representation of the liver lesions could be investigated on a larger data set.

## Conclusion

The proposed classification system, based on features derived from clinical DCE-MR and T2-weighted images, as well as risk factors, is able to classify five common focal liver lesion types (adenoma, cyst, hemangioma, HCC, and metastasis) with an overall accuracy of 0.77, and to differentiate between benign and malignant lesions with an overall accuracy of 0.90. This is a step forward to a clinically useful aid for focal liver lesion diagnosis.

## Supporting information

S1 File Feature values of all lesions. (XLSX)
